# Therapeutic blockade of HMGB1 reduces early motor deficits, but not survival in the SOD1^G93A^ mouse model of amyotrophic lateral sclerosis

**DOI:** 10.1186/s12974-019-1435-2

**Published:** 2019-02-19

**Authors:** John D. Lee, Ning Liu, Samantha C. Levin, Lars Ottosson, Ulf Andersson, Helena E. Harris, Trent M. Woodruff

**Affiliations:** 1 Faculty of Medicine, School of Biomedical Sciences, The University of Queensland, St Lucia, Brisbane, QLD 4072 Australia; 2 Faculty of Medicine, University of Queensland Centre for Clinical Research, The University of Queensland, Herston, Brisbane, QLD 4029 Australia; 3 Department of Women’s and Children’s Health, Karolinska Institutet, Stockholm, Sweden; 4 Centre for Molecular Medicine, Department of Medicine, Karolinska Institutet, Stockholm, Sweden

**Keywords:** TLR4, RAGE, Neuroinflammation, Innate immune system

## Abstract

**Background:**

Amyotrophic lateral sclerosis (ALS) is a fatal and rapidly progressing neurodegenerative disease without effective treatment. The receptor for advanced glycation end products (RAGE) and the toll-like receptor (TLR) system are major components of the innate immune system, which have been implicated in ALS pathology. Extracellularly released high-mobility group box 1 (HMGB1) is a pleiotropic danger-associated molecular pattern (DAMP), and is an endogenous ligand for both RAGE and TLR4.

**Methods:**

The present study examined the effect of HMGB1 inhibition on disease progression in the preclinical SOD1^G93A^ transgenic mouse model of ALS using a potent anti-HMGB1 antibody (2G7), which targets the extracellular DAMP form of HMGB1.

**Results:**

We found that chronic intraperitoneal dosing of the anti-HMGB1 antibody to SOD1^G93A^ mice transiently improved hind-limb grip strength early in the disease, but did not extend survival. Anti-HMGB1 treatment also reduced tumour necrosis factor α and complement C5a receptor 1 gene expression in the spinal cord, but did not affect overall glial activation.

**Conclusions:**

In summary, our results indicate that therapeutic targeting of an extracellular DAMP, HMGB1, improves early motor dysfunction, but overall has limited efficacy in the SOD1^G93A^ mouse model of ALS.

## Background

Amyotrophic lateral sclerosis (ALS) is an adult onset neurodegenerative disease, which is characterised by the irreversible loss of upper and lower motor neurons in the motor cortex, brainstem and spinal cord. This selective loss of neurons leads to muscle denervation, and atrophy, resulting in paralysis and eventual death via respiratory muscle failure [1]. The mechanisms underlying ALS pathogenesis are still unclear, but an emerging body of evidence suggests that immune and inflammatory factors could contribute to the progression of the disease [2–4].

The receptor for advanced glycation end products (RAGE), the toll-like receptor (TLR) system and the complement C5a receptor 1 (C5aR1) are major components of the innate immune system, which have been implicated in ALS pathology. RAGE and TLR4 are generally considered pro-inflammatory receptors expressed by numerous immune and non-immune cells, including cells within the central nervous system (CNS) [5, 6]. Multiple studies have demonstrated that inhibition and/or genetic deletion of RAGE, TLR4, or C5aR1 has beneficial effects on survival and disease progression in animal models of ALS [6–11], suggesting that these immune receptors play a pathogenic role in the disease.

Extracellularly released high-mobility group box 1 (HMGB1) is a pleiotropic danger-associated molecular pattern (DAMP), and is an endogenous ligand for both RAGE and TLR4. HMGB1 is passively released by damaged cells or secreted from activated immune cells into the extracellular milieu, driving inflammatory response in numerous inflammatory diseases, and can be blocked by antibodies specific for the extracellular DAMP form of HMGB1. RAGE and TLR4 activation through the disulfide form of HMGB1 can induce neuroinflammation by releasing cytokines such as tumour necrosis factor-α and interleukins, which have been shown to be involved in ALS pathogenesis [7, 12]. Extracellular HMGB1 can also bind to DNA, lipopolysaccharide and many other immune-activating molecules such as cytokines (IL-1α and IL-1β), which can initiate and mediate inflammatory responses by allowing interactions with a greater number of pro-inflammatory cytosolic receptors [13–15]. Importantly, HMGB1 has been shown to translocate from the nucleus to the cytoplasm in reactive astrocytes and microglia in ALS patients and mouse models [6, 16, 17], suggesting a potential pathogenic role for HMGB1 in ALS.

Since RAGE, TLR4 and HMGB1 are all upregulated in ALS, we hypothesised that therapeutic targeting of the extracellular HMGB1 could be neuroprotective in this disease. To test this, we examined the effect of HMGB1 inhibition on disease progression in the preclinical SOD1^G93A^ transgenic mouse model of ALS using a potent anti-HMGB1 antibody (2G7), which targets the extracellular DAMP form of HMGB1. We found that chronic intraperitoneal dosing of the anti-HMGB1 antibody to SOD1^G93A^ mice transiently improved hind-limb grip strength early in the disease process, but did not extend survival. Anti-HMGB1 treatment also reduced TNFα and C5aR1 gene expression in the spinal cord, but did not affect overall glial activation. In summary, our results indicate that therapeutic targeting of extracellular DAMP, HMGB1 signalling protects against early motor dysfunction, but overall has limited efficacy in the SOD1^G93A^ mouse model of ALS.

## Methods

### Animals

Transgenic SOD1^G93A^ mice (B6-Cg-Tg (SOD1-G93A) 1Gur/J) expressing the high copy number (~ 25 copies) of mutant human SOD1 on a C57BL/6J background were initially obtained from Jackson laboratory (Bar Harbor, ME, USA). A breeding colony was maintained at the University of Queensland Biological Resources Animal Facilities under specific pathogen-free conditions. For all therapeutic efficacy studies, female SOD1^G93A^ littermates (i.e. paired mice from the same litter) were used and separated using a simple randomisation procedure (coin toss) to receive either isotype control antibody, or anti-HMGB1 antibody treatment, in a blinded manner. Transgene copy number for SOD1^G93A^ mice was verified by quantitative PCR as previously described [18]. All animals were group housed (2–3 mice/cage) under identical conditions in a 12 h light/dark cycle (lights on at 0630) with free access to food and water.

### Anti-HMGB1 antibody treatment

Monoclonal humanised anti-HMGB1 antibody (clone 2G7, IgG2b) has previously been characterised to show neutralising activity of HMGB1 [19]. For early treatment, anti-HMGB1 antibody (produced from a hybridoma at Karolinska Institutet) was administered weekly to mice via intraperitoneal injection (100 μg/mouse injection). Litter-matched female SOD1^G93A^ transgenic mice were administered with control IgG2b (vehicle; Innovagen, Lund, Sweden) or anti-HMGB1 antibody prophylactically from 35 days postnatal (termed ‘pre-onset’). For therapeutic treatment, anti-HMGB1 antibody or control IgG2b were administered from 70 days postnatal, which is the age where initial motor deficit symptoms are present (termed ‘post-onset’; [10, 20]). These treatments were continued weekly throughout until the end-stage of disease (i.e. point of euthanasia for survival). Another cohort of animals was treated with control IgG2b or anti-HMGB1 antibody from 35 days till 133 days postnatal (termed ‘mid-symptomatic stage’), where spinal cord and skeletal muscles were collected for quantitative PCR and immunohistochemistry analysis to measure the degree of inflammation. To remove any potential bias, all antibody and vehicle treatments were coded, and then administered and subsequently analysed by a researcher (JDL) blinded to the treatment groups. Blinding was conducted in a manner such that neither the experimenter, nor the entire research team were aware of the treatment code. De-coding only occurred after all animal experiments were completed.

### Survival analysis and motor score

Survival was determined by the inability of the animal to right itself within 15–30 s if laid on either side. This is a widely accepted endpoint for life span studies in ALS mice [21, 22] and guarantees that euthanasia occurs prior to the mice being unable to reach food or water. A motor score was assigned to each mouse weekly, reflecting their motor function based on the presentation of hind-limb tremor, gait abnormalities, hind-limb splay, hind-limb paralysis and presence of the righting reflex [23]. Each factor was assigned a value of 0 or 1, corresponding to normal and abnormal phenotype respectively. An exception to this was the hind-limb splay and righting reflex, which ranged from 0, 1 and 2, coinciding with normal splay and 0 s, partially collapsed to the lateral midline and below 5 s and completely collapsed to the lateral midline and greater than 15 s for hind-limb splay and righting reflex time respectively. Each parameter was scored and summated to calculate the motor score for each mouse (Table 1).

**Table 1:** Summary of phenotypic observations used to assign motor scores

Phenotype	Score 0	Score 1	Score 2
Hind-limb tremor	Normal	Tremor	N/A
Gait abnormalities	Normal	Gait abnormalities	N/A
Hind-limb splay	Normal	Partially collapsed to the lateral midline	Completely collapsed to the lateral midline
Hind-limb paralysis	Normal	Dragging hind-limbs	N/A
Righting reflex	0 s	< 5 s	> 15 s

### Weight measurements and hind limb grip strength test

Isotype control and anti-HMGB1 antibody-treated SOD1^G93A^ mice were weighed weekly at the same time of day (1600–1800 h), from 42 days of age until the defined end-stage (loss of righting reflex). A digital force gauge (Ugo Basile) was used to measure maximal hind-limb muscle grip strength. Mice were held by their tail and lowered until their hind limbs grasped the T-bar connected to the digital force gauge. The tail was then lowered until the body was horizontal with the apparatus, and mice gently pulled away from the T-bar with a smooth steady motion until both of their hind limbs released the bar. The strength of the grip was measured in gram force. Each mouse was given ten attempts and the maximum grip strength from these attempts recorded [20].

### Tissue preparation for microglia/astrocyte quantification and immunohistochemistry

Isotype control or anti-HMGB1 antibody-treated SOD1^G93A^ mice at mid-symptomatic stage (*n* = 4 per treatment group) were euthanized by intraperitoneal injection of zolazapam (50 mg/kg; Zoletil, Lyppard) and xylazine (10 mg/kg; Xylazil, Lyppard). Mice were then fixed by transcardiac perfusion with 2% sodium nitrite in 0.1 M phosphate buffer (pH 7.4; Sigma-Aldrich, St Louis, MO, USA) followed by 4% paraformaldehyde in 0.1 M phosphate buffer (4% PFA-PB, pH 7.4; Sigma-Aldrich, St Louis, MO, USA). Lumbar spinal cords were collected and placed into 4% PFA-PB for 2 h at 4 °C. Following this incubation, spinal cords were washed 3 × 5 min in phosphate-buffered saline (PBS; pH 7.4), followed by submersion in sucrose solution at 15% then 30% in PBS (pH 7.4). Lumbar spinal cords were then embedded in optimal cutting temperature compound (Sakura, Finetek, Torrance, CA, USA) then snap frozen in liquid nitrogen. Lumbar spinal cords were sectioned into 16-μm-thick transverse and coronal sections and dry mounted onto Superfrost Plus slides (Menzel-Glaser, Braunschweig, Germany) for quantitation of astrocytes and microglia as detailed below.

### Estimation of astrocytes and microglia

For estimation of astrocytes and microglia within the lumbar spinal cord, sections were rehydrated in PBS (pH 7.4) then blocked in PBS containing 3% bovine serum albumin (BSA) for 1 h at room temperature. Sections were incubated overnight at 4 °C with the astrocyte (mouse anti-GFAP; 1:1000, BD Biosciences, San Diego, CA, USA) and microglia (rat anti-CD11b; 1:500, Abcam, Cambridge, MA, USA) markers. Sections were washed with PBS for 3 × 10 min prior to incubation overnight at 4 °C with the Alexa secondary cocktail: Alexa Fluor 555 dye-conjugated goat anti-rat (1:1000, Invitrogen, Eugene, OR, USA) and Alexa Fluor 488 dye-conjugated goat anti-mouse (1:600, Invitrogen, Eugene, OR, USA) antibody. All primary and secondary antibodies were diluted in PBS (pH 7.4) containing 1% BSA. Sections were then washed for 3 × 5 min in PBS, then mounted with Prolong Gold Anti-Fade medium containing 4,6-diamidino-2-phenylindole (DAPI; Invitrogen, Eugene, OR, USA). Quantification of GFAP and CD11b immunostaining was performed on ~ 11 to 14 lumbar spinal cord sections spaced 320 μm apart and expressed as the percentage immunoreactive area per section [24]. Quantification was performed within the second lumbar dorsal root ganglia (L2) to the fifth lumbar dorsal root ganglia (L5), selected with the aid of the mouse spinal cord atlas [25]. Staining procedures and image exposures were all standardised between treatment groups and between sections. The treatment groups were not made available to the researchers until the completion of the study.

### Quantification of activated microglia numbers

The cell body of microglia was labelled with the nuclear marker, DAPI. As microglia are known to display morphological changes when they become activated, such as an increase in cell body size, thickening of proximal processes and a decrease in the ramification of distal branches [26], activated microglia were defined by (i) the presence of one DAPI stain, (ii) an amoeboid cell body and (iii) proximal processes length ≤ 1–2 μm [27]. The total number of activated microglia was determined by the average of 11–14 sections, with the overall average multiplied by the number of sections within L2–L5 regions. The treatment groups were not made available to the researchers until quantification was completed.

### Real-time quantitative PCR

Total RNA was isolated from lumbar spinal cord, gastrocnemius and tibialis anterior muscle of isotype control and anti-HMGB1 antibody-treated SOD^G93A^ mice using an RNeasy Lipid Tissue extraction kit according to manufacturer’s instructions (QIAGEN, CA, USA). Total RNA was purified from genomic DNA contamination using Turbo DNAse treatment (Ambion, NY, USA), then converted to cDNA using AffinityScript cDNA synthesis kit according to manufacturer’s instructions (Agilent Technologies, CA, USA). Commercially available gene-specific Taqman probes for integrin alpha M (Itgam; Mm00434455_m1), CD68 antigen (CD68; Mm03047343_m1), allograft inflammatory factor 1 (Aif1; Mm00479862_g1), lymphocyte antigen 6 complex, locus C1 (Ly6c1/Ly6c2; Mm03009946_m1), glial fibrillary acidic protein (Gfap; Mm01253033_m1), tumour necrosis factor (Tnf; Mm00443258_m1), interleukin 1 beta (Il1b; Mm00434228_m1), advanced glycosylation end product-specific receptor (Ager; Mm01134790_g1), complement component 5a receptor 1 (C5ar1; Mm00500292_s1) and toll-like receptor 4 (Tlr4; Mm00445273_m1) were used to amplify target gene of interest (Applied Biosystems, MA, USA). Relative target gene expression to geometric mean of reference genes glyceraldehyde-3-phosphate dehydrogenase (Gapdh; Mm99999915_g1) and beta actin (Actb; Mm02619580_g1) was determined using this formula: 2^-∆CT^ where ∆CT = (Ct _(target gene)_ – Ct _(Gapdh and Actb)_), as per our previous studies [24, 28]. Final measures are presented as relative levels of gene expression in anti-HMGB1 antibody-treated SOD1^G93A^ mice compared with expression in isotype control-treated mice. Probe sets were tested over a serial cDNA concentration for amplification efficiency. No reverse transcription, and water as no template control, was used as negative controls. All samples were run in triplicate and were tested in three separate experiments.

### Statistical analysis

All analyses were performed using GraphPad Prism 7.0 (San Diego, CA, USA). The statistical difference for survival analyses between isotype control and anti-HMGB1 antibody-treated SOD1^G93A^ mice were analysed using log rank (Mantel-Cox) test. The statistical difference between isotype control and anti-HMGB1 antibody-treated SOD1^G93A^ mice for body weight, hind-limb grip strength and motor score were analysed using a two-way ANOVA and a post-hoc Bonferroni’s multiple comparisons test for each time point. For the results from GFAP and CD11b quantification and quantitative real-time PCR, statistical difference between isotype control and anti-HMGB1 antibody-treated SOD1^G93A^ mice were determined using two-tailed student *t* test. All data are presented as mean ± SEM and the differences were considered significant when *P* < 0.05.

## Results

### Pre-onset anti-HMGB1 antibody treatment transiently improves hind-limb grip strength but does not extend survival in SOD1^G93A^ mice

**Fig. 1:**
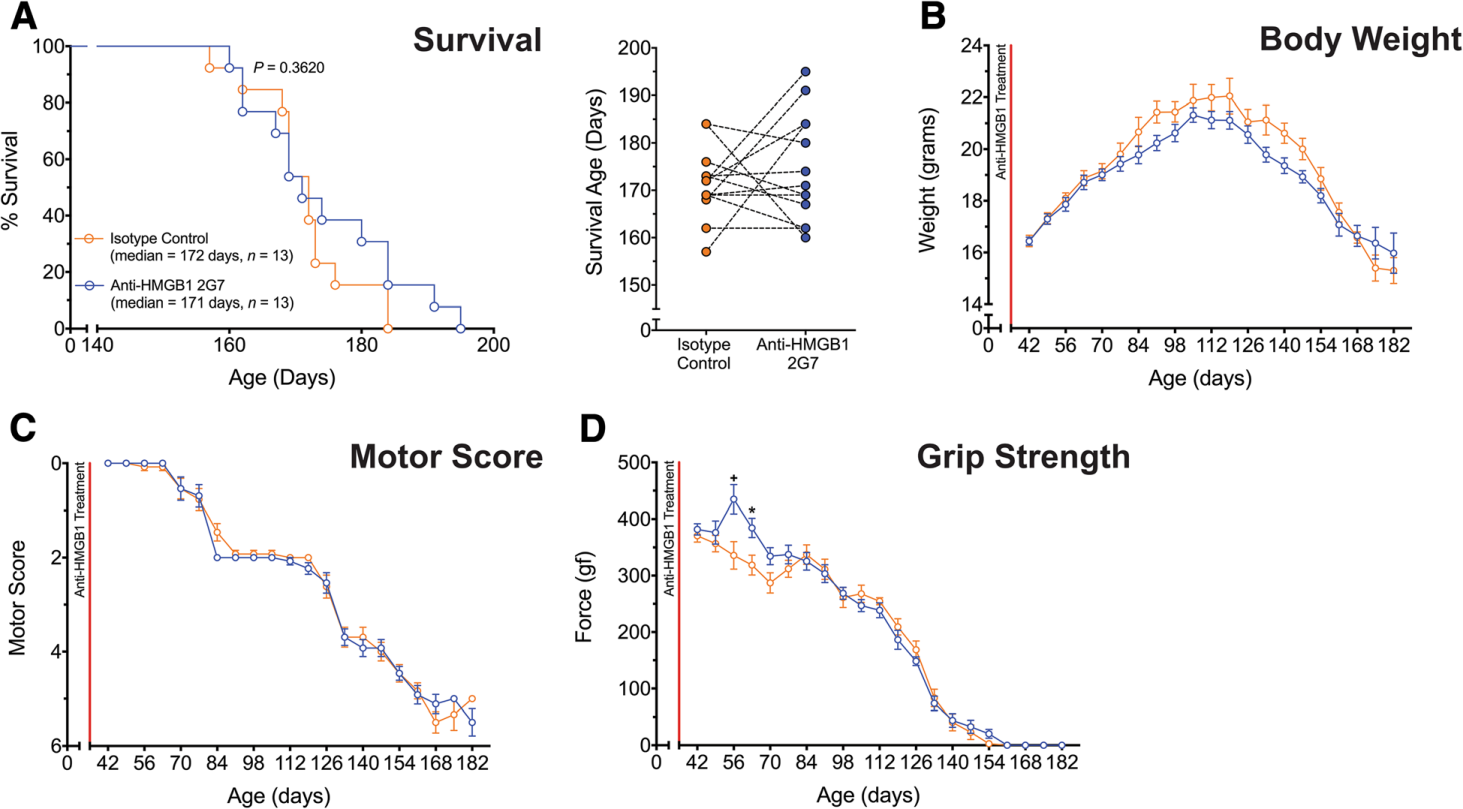
Pre-onset anti-HMGB1 2G7 treatment improves early hind-limb grip strength deficit, but does not extend survival in SOD1^G93A^ transgenic mice. SOD1^G93A^ mice were intraperitoneally injected weekly with the anti-HMGB1 antibody at 35 days of age (red line). **a** Left panel shows a Kaplan-Meier plot of ages (in days) in which SOD1^G93A^ mice treated with isotype control (vehicle, 100 μg; orange line) or anti-HMGB1 antibody (100 μg; blue line) reached end-stage of disease (complete hind-limb paralysis and an inability to right itself once placed on its back; *n* = 13, *P* = 0.362, log-rank test). Anti-HMGB1 treatment at 35 days of age (100 μg) resulted in no extension in survival time compared with vehicle treatment. **a** Right panel shows the end-stage survival age for each litter-matched pair of vehicle- and anti-HMGB1-treated SOD1^G93A^ mice. **b**, **c** Shows no differences in body weight and motor score between vehicle (orange line) and anti-HMGB1 (blue line) treated SOD1^G93A^ mice (*n* = 13, *P* > 0.05, two-way ANOVA). **d** Shows an early transient improvement in hind-limb grip strength for anti-HMGB1-treated versus vehicle-treated SOD1^G93A^ mice at 56 and 63 days of age (*n* = 13, *p < 0.05, +*p* < 0.0001, two-way ANOVA with post-hoc Bonferroni’s multiple comparisons test). Data are expressed as mean ± SEM

In the first treatment study, a cohort of litter-matched SOD1^G93A^ mice was administered isotype control, or anti-HMGB1 antibody treatment from 35 days of age onwards. At this age, SOD1^G93A^ mice do not have any motor neuron loss [20]. SOD1^G93A^ mice treated with anti-HMGB1 antibody from this ‘pre-onset’ age had no significant extension in survival time when compared with litter-matched untreated SOD1^G93A^ mice (*p* = 0.3620, *n* = 13; Fig. 1a). There was also no difference in body weight loss between isotype control and anti-HMGB1 antibody-treated SOD1^G93A^ mice (*p* > 0.05, *n* = 13; Fig. 1b). Motor deficits were also assessed in these animals using motor score and hind-limb grip strength. Anti-HMGB1 antibody treatment showed no improvement in motor scores when compared to control antibody-treated SOD1^G93A^ mice (*p* > 0.05, *n* = 13; Fig. 1c); however, anti-HMGB1 antibody treatment significantly counteracted the loss of hind limb grip strength earlier in the disease at 56 and 63 days of age when compared to control antibody-treated SOD1^G93A^ mice (*p < 0.05, + *p* < 0.0001, *n* = 13; Fig. 1d).

### Post-onset anti-HMGB1 antibody treatment does not extend survival or improve motor performance in SOD1^G93A^ mice

**Fig. 2:**
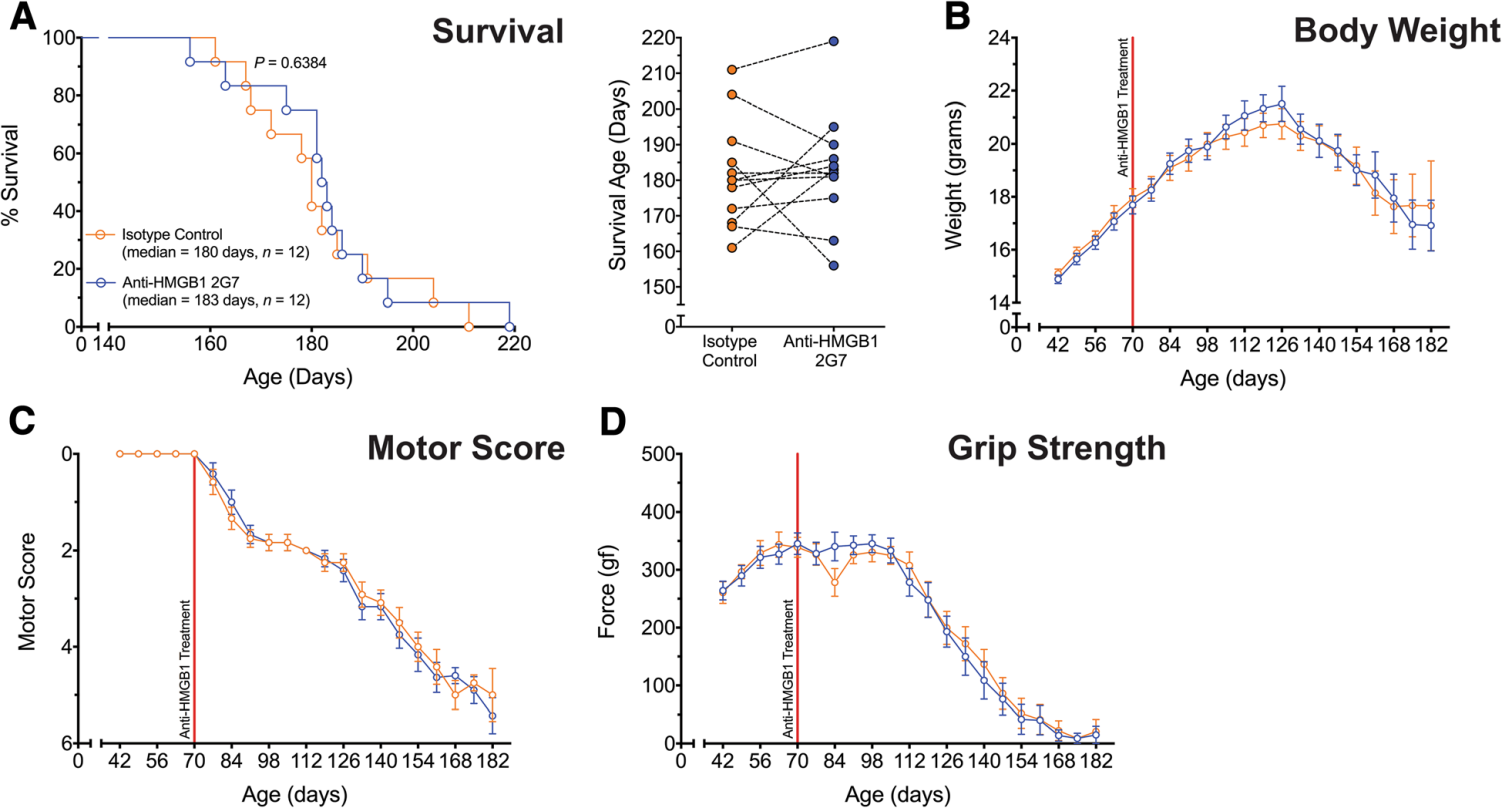
Post-onset anti-HMGB1 2G7 treatment has no effect on disease in SOD1^G93A^ transgenic mice. SOD1^G93A^ mice were intraperitoneally injected weekly with the anti-HMGB1 antibody at 70 days of age (red line). **a** Left panel shows a Kaplan-Meier plot of ages (in days) in which SOD1^G93A^ mice treated with isotype control (vehicle, 100 μg; orange line) or anti-HMGB1 antibody (100 μg; blue line) reached end-stage of disease (complete hind-limb paralysis and an inability to right itself once placed on its back; *n* = 12, *p* = 0.6384, log-rank test). **a** Right panel shows the end-stage survival age for each litter-matched pair of vehicle- and anti-HMGB1-treated SOD1^G93A^ mice, demonstrating no differences in survival time between the groups. **b**–**d** Panels show no difference in body weight (**b**), motor score (**c**) and hind-limb grip strength (**d**) between vehicle (orange line) and anti-HMGB1 (blue line) treated SOD1^G93A^ mice (*n* = 12, *p* > 0.05, two-way ANOVA). Data are expressed as mean ± SEM

We next determined if HMGB1 inhibition at a later stage of disease could reduce ALS pathology in mice. SOD1^G93A^ mice were therefore treated with anti-HMGB1 antibody (100 μg) at 70 days of age, when there is considerable decline in motor performance and motor neuron loss in the SOD1^G93A^ mouse model [20]. Anti-HMGB1 antibody treatment in SOD1^G93A^ mice from this post-onset disease age showed no change in survival time compared to litter-matched control antibody-treated SOD1^G93A^ mice (*p* = 0.6384, *n* = 12; Fig. 2a). Similar to the pre-onset treatment group, post-onset anti-HMGB1 antibody treatment did not affect body weight loss in SOD1^G93A^ mice (*p* > 0.05, *n* = 12; Fig. 2b). Post-onset anti-HMGB1 antibody treatment also did not significantly improve motor score and hind-limb grip strength loss in SOD1^G93A^ mice (*p* > 0.05, *n* = 12; Fig. 2c and d).

### Anti-HMGB1 antibody treatment does not alter microglia and astrocytes in the spinal cord of SOD1^G93A^ mice

**Fig. 3:**
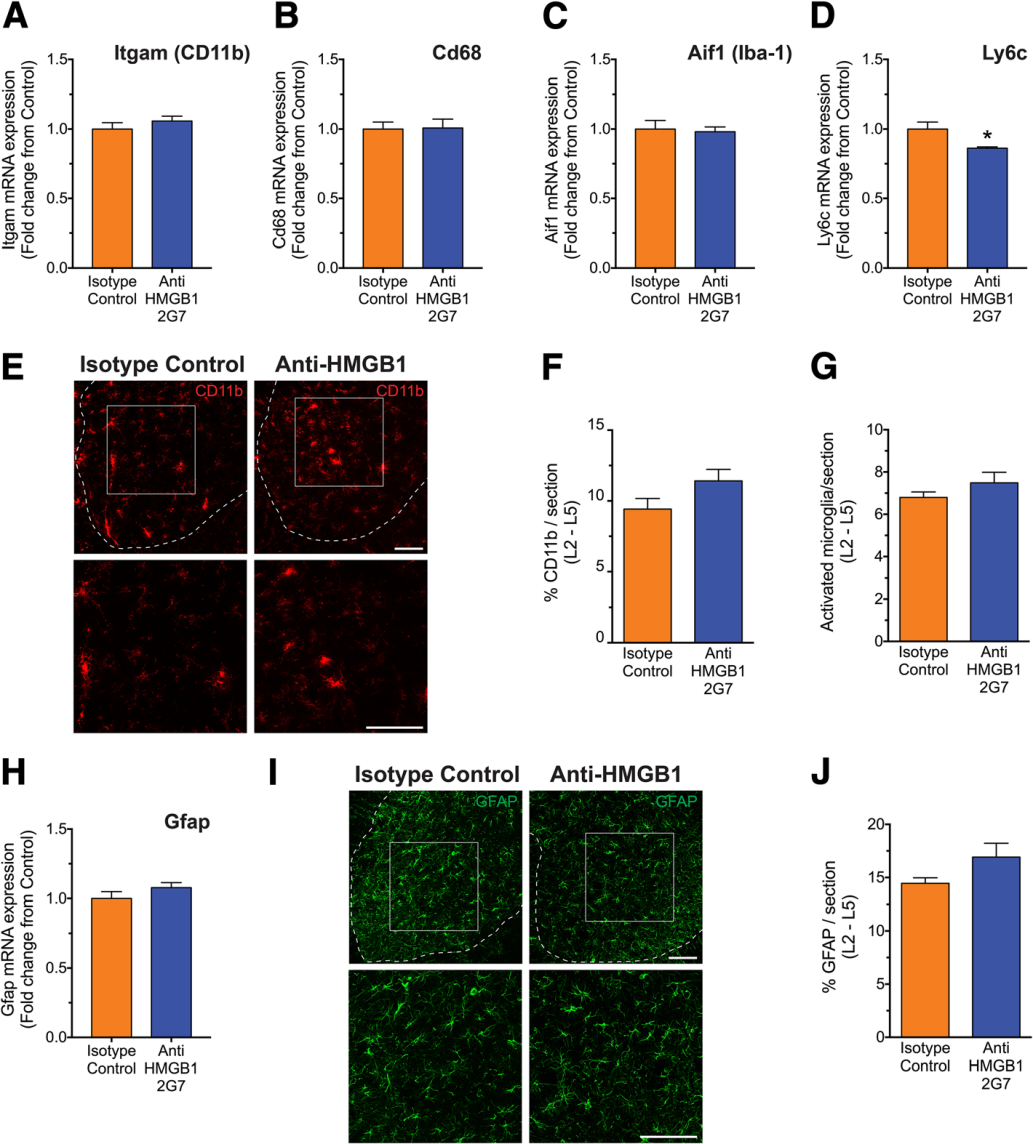
No change in microglia and astrocyte markers in lumbar spinal cord between isotype control and anti-HMGB1 antibody treated SOD1^G93A^ mice. SOD1^G93A^ mice were intraperitoneally injected weekly with the anti-HMGB1 antibody at 35 days of age (100 μg). Major non-neuronal cell populations (microglia/monocytes and astrocytes) in vehicle and anti-HMGB1-treated SOD1^G93A^ mice were investigated at mid-symptomatic stage of disease (133 days) using quantitative PCR and immunohistochemistry. **a**–**c** Shows anti-HMGB1 treatment had no effect on microglia (Itgam, Cd68 and Aif1) mRNA transcript levels (*n* = 6, *p* > 0.05, Student’s *t* test). **d** Shows a reduction in monocyte (Ly6c) mRNA transcript levels in anti-HMGB1-treated SOD1^G93A^ mice when compared to isotype control-treated SOD1^G93A^ mice (*n* = 6, * *P* < 0.05, Student’s *t* test). **e** Shows representative images of CD11b-positive microglia in the lumbar spinal cord of isotype control and anti-HMGB1-treated SOD1^G93A^ mice at 133 days of age. Dashed line shows the outline of the ventral horn with higher magnification of the white square. Scale bar = 100 μm. **f**, **g** Shows no change in microglia expression and activated microglia (amoeboid) in anti-HMGB1-treated SOD1^G93A^ mice compared with isotype control-treated SOD1^G93A^ mice (*n* = 4, *p* > 0.05, Student’s *t* test). **h** Shows no change in astrocyte (Gfap) mRNA transcript levels between isotype control and anti-HMGB1-treated SOD1^G93A^ mice (*n* = 6, *p* > 0.05, Student’s *t* test). **i** Show representative images of GFAP-positive astrocytes in the lumbar spinal cord of isotype control and anti-HMGB1-treated SOD1^G93A^ mice at 133 days of age. Dashed line shows the outline of the ventral horn with higher magnification of the white squares. Scale bars = 100 μm. **j** Shows no change in astrocyte expression in anti-HMGB1 treated-SOD1^G93A^ mice compared with isotype control-treated SOD1^G93A^ mice (*n* = 4, *p* > 0.05, Student’s *t* test). Data are presented as mean ± SEM

Given previous studies demonstrating a potential role of microglia and astrocytes during disease progression of ALS [29–32], and the potential role for HMGB1 in modulating gliosis [33], we also examined glial markers in the pre-onset treatment group. We first investigated whether inhibition of HMGB1 in SOD1^G93A^ mice had any effect on microglia and astrocytes in the lumbar spinal cord. mRNA expression levels of Itgam, Cd68 and Aif1 (markers of both resident microglia and infiltrating monocyte/macrophages) and Ly6c (predominant marker of early infiltrating monocyte/macrophages) were measured in the lumbar spinal cord of isotype control and anti-HMGB1 antibody-treated SOD1^G93A^ mice at mid-symptomatic stage of disease progression using quantitative real-time PCR. Itgam, Cd68 and Aif1 transcripts were unaltered in anti-HMGB1 antibody-treated SOD1^G93A^ mice when compared to control antibody-treated SOD1^G93A^ mice (*n* = 6, *p* > 0.05; Fig. 3a–c). However, Ly6c transcripts were significantly reduced in anti-HMGB1 antibody-treated SOD1^G93A^ mice when compared to control antibody-treated SOD1^G93A^ mice (*n* = 6, *p < 0.05; Fig. 3d). Microglial activation was also examined using immunofluorescence. No change in immunoreactive area of CD11b-positive microglia, and the number of activated microglia in the lumbar spinal cord of control or anti-HMGB1 antibody-treated SOD1^G93A^ mice were found at mid-symptomatic stage of disease (*n* = 4, *p* > 0.05; Fig. 3e, f). Next, we investigated the mRNA expression levels of Gfap (marker of astrocytes) in the lumbar spinal cord of isotype control and anti-HMGB1 antibody-treated SOD1^G93A^ mice at mid-symptomatic stage of disease. Gfap transcript was also unaltered in anti-HMGB1 antibody-treated SOD1^G93A^ mice when compared to control antibody-treated SOD1^G93A^ mice (*n* = 6, *p* > 0.05; Fig. 3g). These results were also confirmed using immunofluorescence, where the immunoreactive area of GFAP-positive astrocytes did not change between control and anti-HMGB1 antibody-treated SOD1^G93A^ mice (*n* = 4, *p* > 0.05; Fig. 3h).

### Anti-HMGB1 antibody treatment reduces TNFα and C5aR1 gene expression in the spinal cord of SOD1^G93A^ mice

**Fig. 4:**
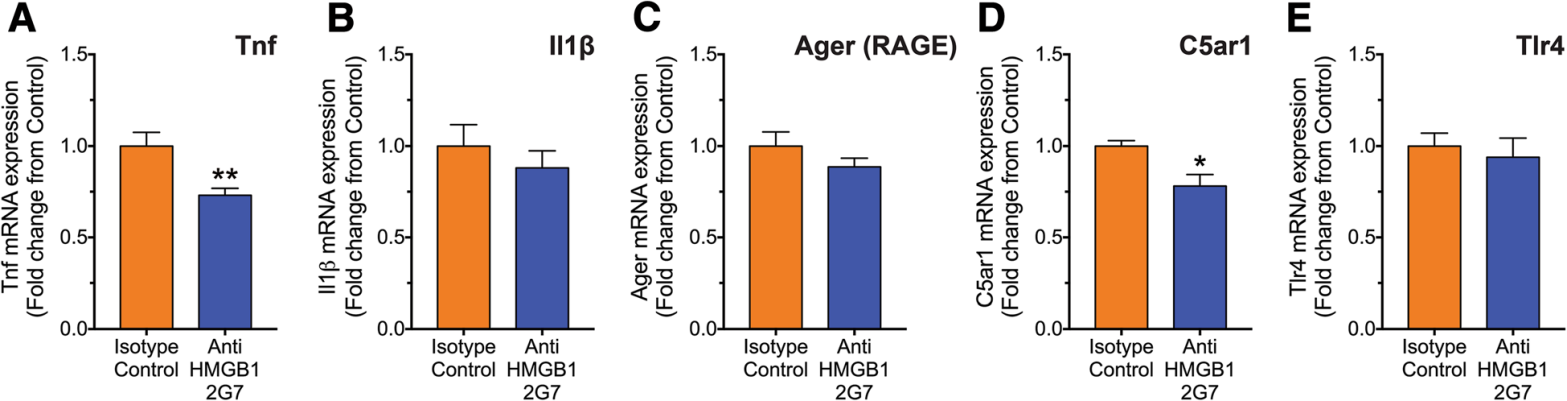
Tnf and C5ar1 transcripts are reduced in lumbar spinal cord of anti-HMGB1 antibody-treated SOD1^G93A^ mice. SOD1^G93A^ mice were intraperitoneally injected weekly with the anti-HMGB1 antibody at 35 days of age (100 μg). Pro-inflammatory cytokines (TNFα and IL-1β) and major innate immune receptors (RAGE, C5aR1 and TLR4) in vehicle and anti-HMGB1-treated SOD1^G93A^ mice was investigated at mid-symptomatic stage of disease (133 days) using quantitative PCR. **a** Show anti-HMGB1 treatment reduces pro-inflammatory cytokine Tnf in the spinal cord of SOD1^G93A^ mice (*n* = 6, **p < 0.01, Student’s *t* test), while no change in Il1β was evident between isotype control and anti-HMGB1-treated SOD1^G93A^ mice (**b**; *n* = 6, *p* > 0.05, Student’s *t* test). Anti-HMGB1 treatment showed slight reduction in C5ar1 mRNA transcript levels while no change was observed for Ager and Tlr4 (**c**–**e**; *n* = 6, *p < 0.05, Student’s *t* test). Data are presented as mean ± SEM

Activation of HMGB1 also induces synthesis of cytokines to modulate inflammatory processes, and has been shown to induce cytokine expression in microglia [34]. Importantly, pro-inflammatory cytokines such as TNFα and IL-1β are thought to propagate disease progression in ALS through the activation of the innate immune system [35]. Hence, we investigated whether inhibition of HMGB1 in SOD1^G93A^ mice had any effect on the expression of TNFα and IL-1β and the several major receptors of the innate immune system (RAGE, complement C5aR1 and TLR4) in the lumbar spinal cord. mRNA expression of Tnf and Il1β was measured in isotype control and anti-HMGB1 antibody-treated SOD1^G93A^ mice at mid-symptomatic stage of disease progression by quantitative real-time PCR. Tnf transcripts were significantly reduced in anti-HMGB1 antibody-treated SOD1^G93A^ mice by 0.27-fold when compared to control antibody-treated SOD1^G93A^ mice (*n* = 6, **p < 0.01; Fig. 4a), while Il1β mRNA expression did not change between control and anti-HMGB1 antibody-treated SOD1^G93A^ mice (*n* = 6, *p* > 0.05; Fig. 4b). There was no change in the mRNA expression of Ager and Tlr4 in the lumbar spinal cord of anti-HMGB1 antibody-treated SOD1^G93A^ mice when compared to control antibody-treated SOD1^G93A^ mice (*n* = 6, *p* > 0.05; Fig. 4c, d). However, C5ar1 mRNA expression was decreased by 0.22-fold in anti-HMGB1 antibody-treated SOD1^G93A^ mice when compared to control antibody-treated SOD1^G93A^ mice (*n* = 6, *p < 0.05; Fig. 4e). Taken together, these results suggest that HMGB1 inhibition reduces certain pro-inflammatory factors in the spinal cord of SOD1^G93A^ mice treated with anti-HMGB1 antibody at mid-symptomatic stage of disease.

### Anti-HMGB1 antibody treatment reduced monocyte markers in the tibialis anterior muscle of SOD1^G93A^ mice

**Fig. 5:**
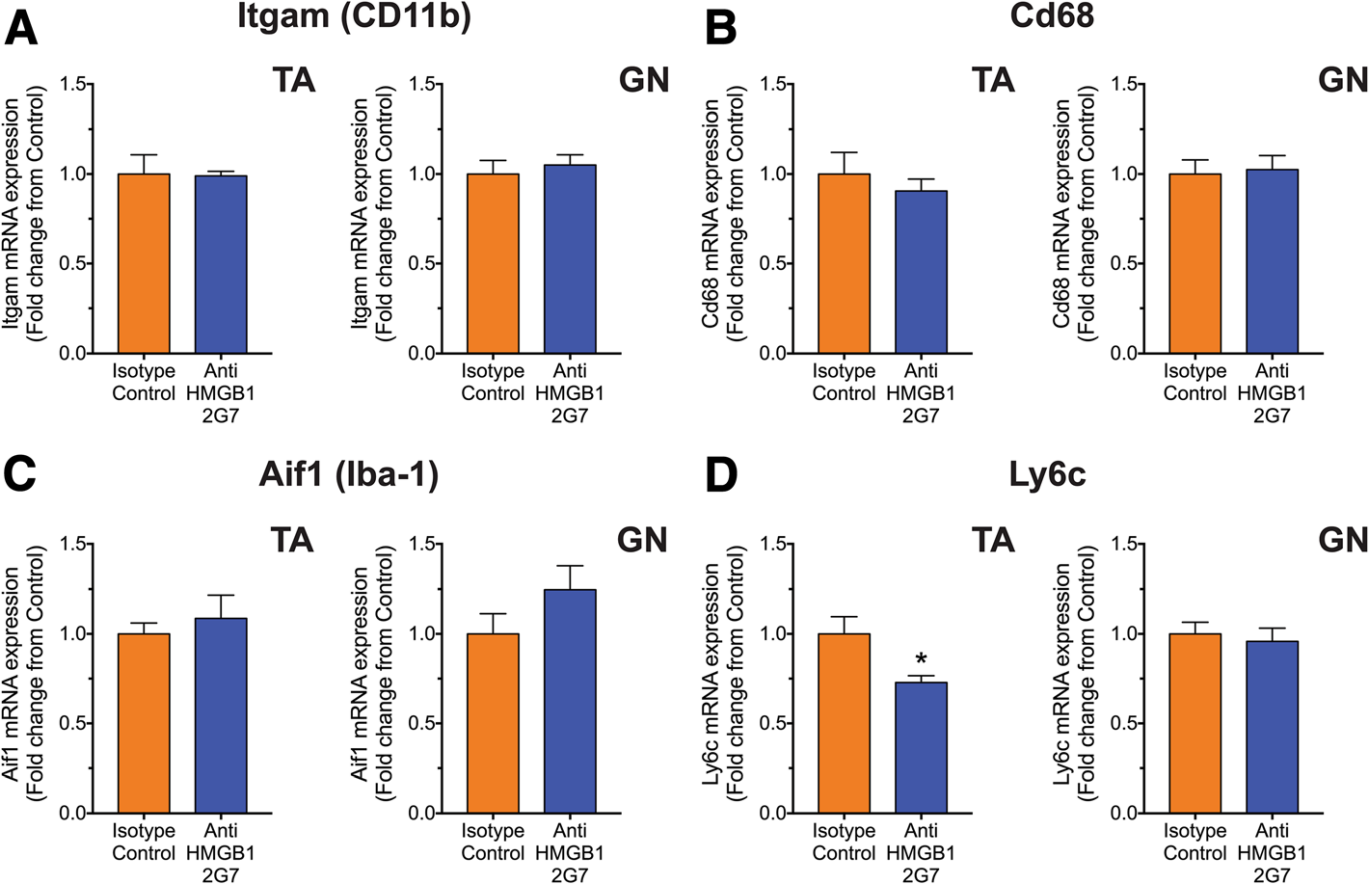
Monocyte and macrophage markers in tibialis anterior and gastrocnemius muscle are not altered between isotype control and anti-HMGB1 antibody-treated SOD1^G93A^ mice. SOD1^G93A^ mice were intraperitoneally injected weekly with the anti-HMGB1 antibody at 35 days of age (100 μg). Monocytes and macrophage markers in tibialis anterior (TA) and gastrocnemius (GN) muscle of vehicle and anti-HMGB1-treated SOD1^G93A^ mice was investigated at mid-symptomatic stage (133 days) using quantitative PCR. **a**–**c** Shows anti-HMGB1 treatment had no effect on macrophage (Itgam, Cd68 and Aif1) mRNA transcript levels in both TA and GN muscles (*n* = 6, *p* > 0.05, Student’s *t* test). **d** Shows a reduction in monocyte (Ly6c) mRNA transcript levels in TA muscle of anti-HMGB1-treated SOD1^G93A^ mice, while no change was evident in GN muscle when compared to isotype control-treated SOD1^G93A^ mice (*n* = 6, *p < 0.05, Student’s *t* test). Data are presented as mean ± SEM

**Fig. 6:**
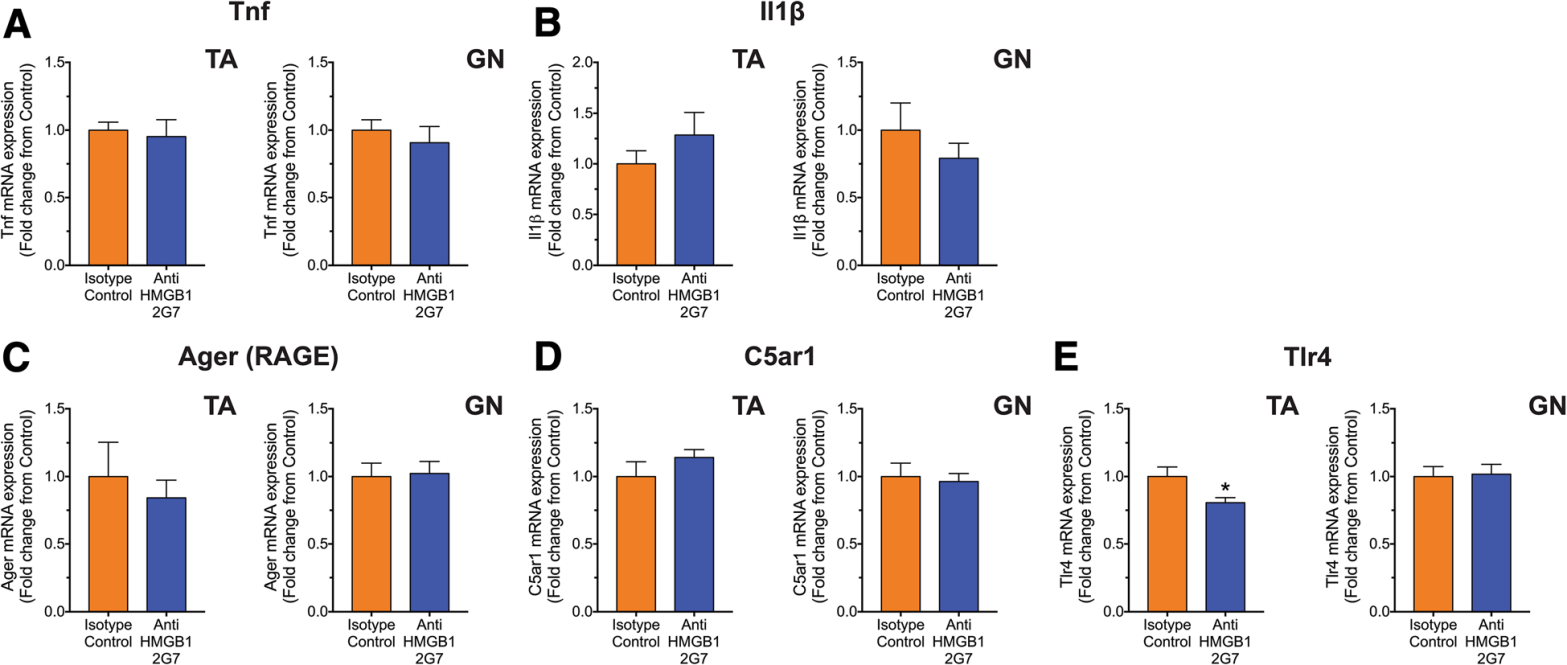
Immune and inflammatory markers are not altered in tibialis anterior and gastrocnemius muscle of anti-HMGB1 antibody-treated SOD1^G93A^ mice. SOD1^G93A^ mice were intraperitoneally injected weekly with the anti-HMGB1 antibody at 35 days of age (100 μg). Pro-inflammatory cytokines (TNFα and IL-1β) and major innate immune receptors (RAGE, C5aR1 and TLR4) in tibialis anterior (TA) and gastrocnemius (GN) muscle of vehicle and anti-HMGB1-treated SOD1^G93A^ mice was investigated at mid-symptomatic stage of disease (133 days) using quantitative PCR. **a**, **b** Shows no change in pro-inflammatory cytokines Tnf and Il1β in both TA and GN muscle of anti-HMGB1-treated SOD1^G93A^ mice when compared to isotype control-treated SOD1^G93A^ mice (*n* = 6, *p* > 0.05, Student’s *t* test). Ager and C5ar1 mRNA transcript levels were not different in isotype control and anti-HMGB1-treated SOD1^G93A^ mice in both TA and GN muscle (**c**, **d**; *n* = 6, *p* > 0.05, Student’s *t* test). While anti-HMGB1 treatment showed a slight reduction in Tlr4 transcript levels in TA muscle, no change was observed in GN muscle of SOD1^G93A^ mice (**e**; *n* = 6 *p < 0.05, Student’s *t* test). Data are presented as mean ± SEM

Given HMGB1’s role as a chemoattractant for leukocytes, and the known role of monocytes/macrophages accumulation in skeletal muscle denervation in SOD1^G93A^ mice [36, 37], we investigated whether neutralising HMGB1 in SOD1^G93A^ mice impacted on peripheral monocytes/macrophages infiltration. mRNA expression levels of Itgam, Cd68, Aif1 (monocytes/macrophage marker) and Ly6c (monocyte marker) were measured in the tibialis anterior (TA) and gastrocnemius (GN) muscles of isotype control and anti-HMGB1 antibody-treated SOD1^G93A^ mice using quantitative real-time PCR. Interestingly, mRNA expression of macrophage markers (Itgam, Cd68 and Aif1) did not change between control and anti-HMGB1 antibody-treated SOD1^G93A^ mice in both TA and GN muscles (*n* = 6, *p* > 0.05; Fig. 5a–c). By contrast, mRNA expression of monocyte marker (Ly6c) was decreased in TA muscle of anti-HMGB1 antibody-treated SOD1^G93A^ mice when compared to control antibody-treated SOD1^G93A^ mice (*n* = 6, *p < 0.05; Fig. 5d). This demonstrates that HMGB1 signalling induces the infiltration of the peripheral monocytes in SOD1^G93A^ mice, which may potentially affect the progression of denervation in these muscles. We also examined the expression of the same panel of inflammatory and innate immune markers as used in the spinal cord, in the skeletal muscles. Interestingly, unlike the spinal cord, anti-HMGB1 antibody treatment did not change cytokines (Tnf and Il1β) or innate immune receptors (Ager and C5ar1) in both TA and GN muscles of SOD1^G93A^ mice but had slight reduction of Tlr4 transcript in TA muscle (*n* = 6, *p < 0.05; Fig. 6a–e).

## Discussion

Although the exact mechanisms that underlie the pathogenesis of ALS remain unclear, there is credible evidence that a co-ordinated action of innate and adaptive immune factors, both in the periphery and the central nervous system, may contribute substantially in the progression of ALS. This includes evidence for major innate immune systems such as the complement cascade at the level of C5a/C5aR1, the TLR system and RAGE, where it is shown that these immune receptors promote neuroinflammation and disease progression of ALS [6–11, 20, 24, 37, 38]. HMGB1 is an ubiquitous nuclear protein that is released extracellularly after cellular stress, damage and death and promotes inflammation by binding to innate immune receptors such as TLR2, TLR4 and RAGE, suggesting that it could play a role in the disease progression of ALS. In support of this, previous studies have demonstrated that TLR2, TLR4 and RAGE are significantly increased on microglia and astrocytes during ALS progression in SOD1^G93A^ mice, and inhibition/ablation of these components have beneficial effects on the disease outcome [6–9, 16]. In addition, we previously demonstrated increased HMGB1, the ligand for TLR2, TLR4 and RAGE, in the spinal cord of SOD1^G93A^ mice, suggesting that there is heightened HGMB1 release and signalling in ALS SOD1^G93A^ mice [6]. In the present study, we extended from these findings by testing the potential efficacy of pharmacological HMGB1 inhibition, before and after disease onset in SOD1^G93A^ mice.

HMGB1 is a highly conserved nuclear protein made up of 215 residues consisting of 2 DNA-binding domains (termed A- and B-boxes) with a highly negatively charged C-terminal tail. The monoclonal anti-HMGB1 2G7 antibody used in our study recognises an epitope in the box A domain and has been previously characterised to show neutralising activity of HMGB1 [39]. Furthermore, this anti-HMGB1 antibody has been shown to neutralise the cytokine isoform of HMGB1 [40]. To determine the effect of HMGB1 neutralisation in SOD1^G93A^ mice disease progression, antibody treatment experiments included mice injected from an early pre-symptomatic age (day 35) to determine the maximum effect of HMGB1 inhibition, as well as at a later onset time point where motor deficits are first evident (day 70). The present study demonstrated that neutralisation of HMGB1 via an intraperitoneal injection of anti-HMGB1 2G7 antibody at both time points did not extend survival time, however transiently improved the early motor deficits and reduced inflammation in the spinal cord of SOD1^G93A^ mice with pre-onset treatment. This is consistent with previous studies using monoclonal anti-HMGB1 2G7 antibody in experimental rodent models of stroke and lupus nephritis where minimal efficacy was observed. For stroke, there was no reduction in infarct volume or improvement in neurological outcomes, following anti-HMGB1 2G7 treatment, although some alleviated sickness behaviour was documented due to reductions in peripheral immune responses [41]. For nephritis, there were no changes in disease parameters including kidney pathology, body weight and proteinuria [42]. However, other studies have demonstrated that HMGB1-blocking therapies show beneficial effects in providing significant protection following traumatic brain injury, arthritis, experimental sepsis and liver injury [19, 43–47]. One explanation for the apparent lack of efficacy of HMBG1 neutralisation in the present study, despite the fact that TLR2, TLR4 and RAGE inhibition in SOD1^G93A^ mice is documented to be beneficial, is that there are other endogenous ligands activating TLR2, TLR4 and RAGE, which could contribute to disease pathology. Indeed, in ALS, other endogenous ligands for these innate immune receptors, such as heat shock protein 60 (HSP60), HSP70 and S100β protein, have been implicated in disease progression of ALS and their inhibition reduces inflammation and ALS disease parameters, suggesting they could contribute to the disease pathogenesis through TLR or RAGE activation. Our results indicate therefore that HMGB1 activation of TLR2, TLR4 and RAGE in SOD1^G93A^ mice may be compensated for by other endogenous ligands, which may explain the lack of efficacy on survival with anti-HMGB1 antibody treatment. Furthermore, others have also suggested that astrocytic HMGB1 signalling in ALS could be neuroprotective via release of neurotrophic factors such as brain-derived neurotrophic factor and glial cell line-derived neurotrophic factor [48].

Anti-HMGB1 antibody treatment also demonstrated no alteration in microglia/macrophages and astrocytes which supports the lack of beneficial effect on disease progression and survival in treated animals. Small reductions in pro-inflammatory cytokines TNFα and innate immune receptors C5aR1 and TLR4 in lumbar spinal cord and skeletal muscle however were observed, indicating that treated mice did have some alterations in inflammatory biomarkers. One limitation of this study is the use of an antibody approach to target HMGB1. Antibodies are known to have limited brain and spinal cord penetration [49], which may have impacted on the potential inhibitory effect of the compound on CNS-derived HMGB1. However, we have previously shown that SOD1^G93A^ mice have a leaky blood-brain barrier/blood-spinal cord barrier early in the disease, which progresses until end-stage [10], and other antibody approaches have been successfully used in SOD1^G93A^ models previously [50]. Furthermore, the reductions in the spinal cord inflammatory markers, C5aR1 and TNFα, suggest that some localised HMGB1 blockade was occurring. Regardless, future investigation of the plasma and CNS pharmacokinetic profile of anti-HMGB1 2G7 antibody is warranted to confirm concentrations in the target tissues following our dosage regime.

## Conclusions

In summary, the present study demonstrated that early neutralisation of extracellularly released HMGB1 with an anti-HMGB1 antibody in SOD1^G93A^ ALS mice transiently improves hind-limb grip strength, associated with reduced spinal cord expression of key pro-inflammatory genes. However, anti-HMGB1 treatment had no effect on motor decline or survival, and did not alter spinal cord glial numbers or activation profiles, suggesting a minimal role for this DAMP in overall neuroinflammation and disease progression. These data therefore indicate that HMGB1 signalling plays a minor role in the SOD1^G93A^ model of ALS, limiting the further exploration of targeted HMGB1 inhibition with antibodies such as 2G7, as a treatment for ALS.

## Abbreviations

ALS: Amyotrophic lateral sclerosis
C5aR1: Complement C5a receptor 1
CNS: Central nervous system
DAMP: Danger-associated molecular pattern
GN: Gastrocnemius muscle
HMGB1: High-mobility group box 1
RAGE: Receptor for advanced glycation end products
TA: Tibialis anterior muscle
TLR: Toll-like receptor
